# The 2006 *Retrovirology *Prize: call for nominations

**DOI:** 10.1186/1742-4690-3-17

**Published:** 2006-03-15

**Authors:** Kuan-Teh Jeang

**Affiliations:** 1 The National Institutes of Health, Bethesda, MD, USA

## Abstract

*Retrovirology *announces a nomination call for its 2006 prize to recognize an outstanding mid-career retrovirologist. The 2005 *Retrovirology *prize was awarded to Dr. Stephen P. Goff.

## 

This month *Retrovirology *completes two years of continuous publishing. At the 24 months juncture, we are pleased with the support and traction that we have achieved within our scientific community. *Retrovirology *is now tracked and indexed in all major bibliographic services including *Medline*, *PubMed*, *Embase*, and *Thomson ISI*; and citations in the literature to *Retrovirology *papers are increasingly numerous.

To our knowledge, we are the only journal focused on retrovirus research that is *Open Access*. Is that important? You bet! When you consider that we are a tightly focused publication serving a numerically small community, and you realize that *Retrovirology *is being accessed over 1740 times each week day and 1670 times each weekend day, then I believe you can appreciate the real demand for and the power of *Open Access*. As science moves increasingly toward globalization, *Retrovirology *embraces the timely and necessary concept that we have a responsibility to distribute scientific knowledge using an access model that transcends professional classifications, national boundaries, individual wealth, and accidents of birth.

In keeping with *Retrovirology's *goal to highlight high quality stringently reviewed science and to bring visibility to retrovirus research, the journal sponsors an annual *Retrovirology *Prize.

## Nominations are being called for the 2006 *Retrovirology *Prize

**Figure 1:**
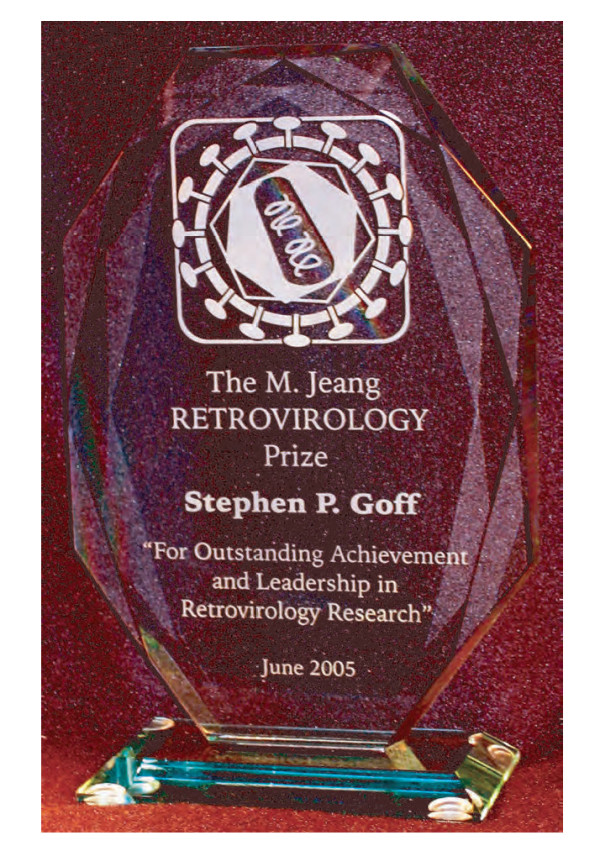
A photograph of the crystal trophy presented to Dr. Stephen P. Goff, winner of the 2005 M. Jeang *Retrovirology *Prize.

**Figure 2:**
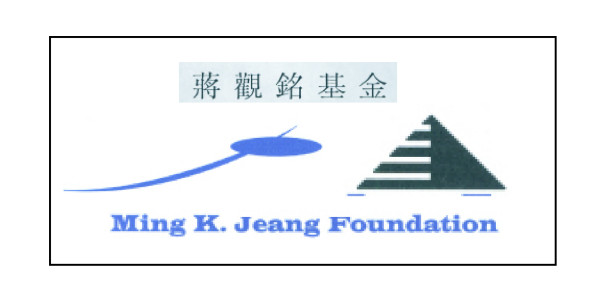
Logo of the Ming K. Jeang Foundation which has made a donation to support the *Retrovirology *Prize.

Last year *Retrovirology *began an annual prize to recognize an outstanding retrovirologist between the ages of 45 to 60 [1]. The *Retrovirology *Prize consists of an attractive crystal trophy (Figure 1), a $3,000 cash award, and a profile article of the winner published in *Retrovirology *about his/her scientific contributions to retrovirus research. The *Retrovirology *Prize is supported in part through a donation from the Ming K. Jeang Foundation (Figure 2), an educational foundation based in Houston, Texas, USA. Accordingly, the Prize is named the M. Jeang *Retrovirology *Prize.

In 2005, Dr. Stephen P. Goff of Columbia University, USA, was our winner [2]. We anticipate selecting an equally outstanding and accomplished scientist for 2006.

## The selection process

As stated previously [1], the Prize alternates yearly between recognizing a non-HIV retrovirologist (2005 and odd years) and an HIV retrovirologist (2006 and even years). There can be some discretion on this criterion exercised from time-to-time by the selection committee. Any individual can initiate a nomination of others or self-nominate. A nomination includes a statement (1000 words or less) of the nominee's significant contributions to retrovirus research; a curriculum vitae of the nominee, and a statement by the nominator that the nominee has agreed to be nominated. The selection committee consists of the Editors of *Retrovirology *(currently, M. Benkirane, B. Berkhout, M. Fujii, K.T. Jeang, M. Lairmore, A. Lever, and M. Wainberg). All nominations submitted to the selection committee must be communicated through an Editorial Board member of *Retrovirology*. Hence, any individual who is not an Editorial board member who wishes to make a nomination should seek out a *Retrovirology *Editorial board member to communicate his/her information to the selection committee. A list of current Editorial Board members can be found at the *Retrovirology *website . Within stipulated age limits, all *Retrovirology *Editors and Editorial Board members are eligible to be nominated with the exception of the Editor-in-Chief who will administer the final selection decision.

For 2006, nominations will begin April 1^st ^and will close June 1^st^. I urge all members of our scientific community to participate in this process for recognizing a deserving colleague.

## References

[B1] Jeang K-T (2005). Life after 45 and before 60: the *Retrovirology *Prize. Retrovirology.

[B2] Jeang K-T (2005). Small philanthropy and big science: the *RETROVIROLOGY *prize and Stephen P. Goff. Retrovirology.

